# Perioperative Doppler measurements of renal perfusion are associated with acute kidney injury in patients undergoing cardiac surgery

**DOI:** 10.1038/s41598-021-99141-y

**Published:** 2021-10-05

**Authors:** Johan Lyngklip Hermansen, Gabriela Pettey, Heidi Tofte Sørensen, Samantha Nel, Nqoba Tsabedze, Arne Hørlyck, Palesa Motshabi Chakane, Henrik Gammelager, Peter Juhl-Olsen

**Affiliations:** 1 Department of Cardiothoracic and Vascular Surgery, Anaesthesia Section, Aarhus University Hospital, Palle Juul-Jensens Boulevard 99, 8200 Aarhus N, Denmark; 2 Department of Clinical Medicine, Aarhus University, Palle Juul-Jensens Boulevard 82, 8200 Aarhus N, Denmark; 3 Department of Anaesthesiology, Charlotte Maxeke Johannesburg Academic Hospital, University of the Witwatersrand, 17 Jubilee Road, Parktown, Johannesburg, 2193 South Africa; 4 Division of Cardiology, Department of Internal Medicine, Charlotte Maxeke Johannesburg Academic Hospital, University of the Witwatersrand, 17 Jubilee Road, Parktown, Johannesburg, 2193 South Africa; 5 Department of Radiology, Aarhus University Hospital, Palle Juul-Jensens Boulevard 99, 8200 Aarhus N, Denmark; 6 Department of Intensive Care, Aarhus University Hospital, Palle Juul-Jensens Boulevard 99, 8200 Aarhus N, Denmark

**Keywords:** Acute kidney injury, Cardiovascular diseases, Clinical trials

## Abstract

Acute kidney injury (AKI) is a frequent and severe complication in cardiac surgery. Normal renal function is dependent on adequate renal perfusion, which may be altered in the perioperative period. Renal perfusion can be assessed with Doppler measurement. We aimed to determine the association between Doppler measurements of renal perfusion and the development of AKI. This was a prospective, observational study of 100 patients with ≥ one risk factor for postoperative AKI undergoing open-heart surgery. Doppler ultrasound examinations were performed before surgery and on the first and fourth postoperative day. AKI was defined according to the KDIGO criteria and subdivided into mild (KDIGO stage 1) and severe AKI (KDIGO stage 2 + 3). Thirty-three patients developed AKI, 25 developed mild and eight developed severe AKI. Abnormal renal venous flow pattern on the first postoperative day was significantly associated with the development of severe AKI (OR 8.54 (95% CI 1.01; 72.2), P = 0.046), as were portal pulsatility fraction (OR 1.07 (95% CI 1.02; 1.13), P = 0.005). Point-of-care Doppler ultrasound measurements of renal perfusion are associated with the development of AKI after cardiac surgery. Renal and portal Doppler ultrasonography can be used to identify patients at high risk or very low risk of AKI after cardiac surgery.

## Introduction

In cardiac surgery, postoperative acute kidney injury (AKI) is a frequent complication occurring in 15–30% of patients^1–3^. Even a slight increase in creatinine is an independent risk factor for increased mortality and morbidity^3,4^. Development of AKI has major consequences for the individual patient, results in prolonged hospitalisation and causes considerably increased expenditure^5^.

The pathophysiology of AKI is multifactorial and involves several mechanisms including renal ischaemia, reperfusion injury and altered perfusion^3,6,7^. However, early diagnosis of postoperative AKI is challenging. Changes in creatinine lag behind results of the actual renal function and the creatinine level may be diluted by cardiopulmonary bypass prime and volume infusion. Furthermore, urine output is not a specific measure of renal function^6,8,9^.

A key element in maintaining normal renal function is the upholding of renal perfusion. Renal perfusion is governed by a complex and not fully understood interplay between volume status, cardiac output and mean arterial pressure. Increasing attention has focused on the importance of the back pressure to venous outflow, which causes a decrease in renal perfusion pressure and renal congestion in case of increased back pressure^10,11^.

Renal perfusion can be assessed with Doppler ultrasound of the renal vasculature allowing for quantification of both renal arterial and renal venous flow characteristics. Hence, these novel ultrasound measures may be able to identify patients suffering from AKI with or without concomitant venous congestion. This may further facilitate appropriate preventive measures and treatment by targeting appropriate renal perfusion. Point-of-care ultrasound examination of patients undergoing cardiac surgery may thus provide valuable information supporting clinical decision-making.

This study investigated the associations and optimal threshold values between perioperative renal Doppler flow measures and AKI following cardiac surgery. We hypothesised that an abnormal renal venous flow category on the first postoperative day is associated with a significantly increased risk of developing AKI after cardiac surgery.

## Methods

### Study design and participants

**Figure 1:**
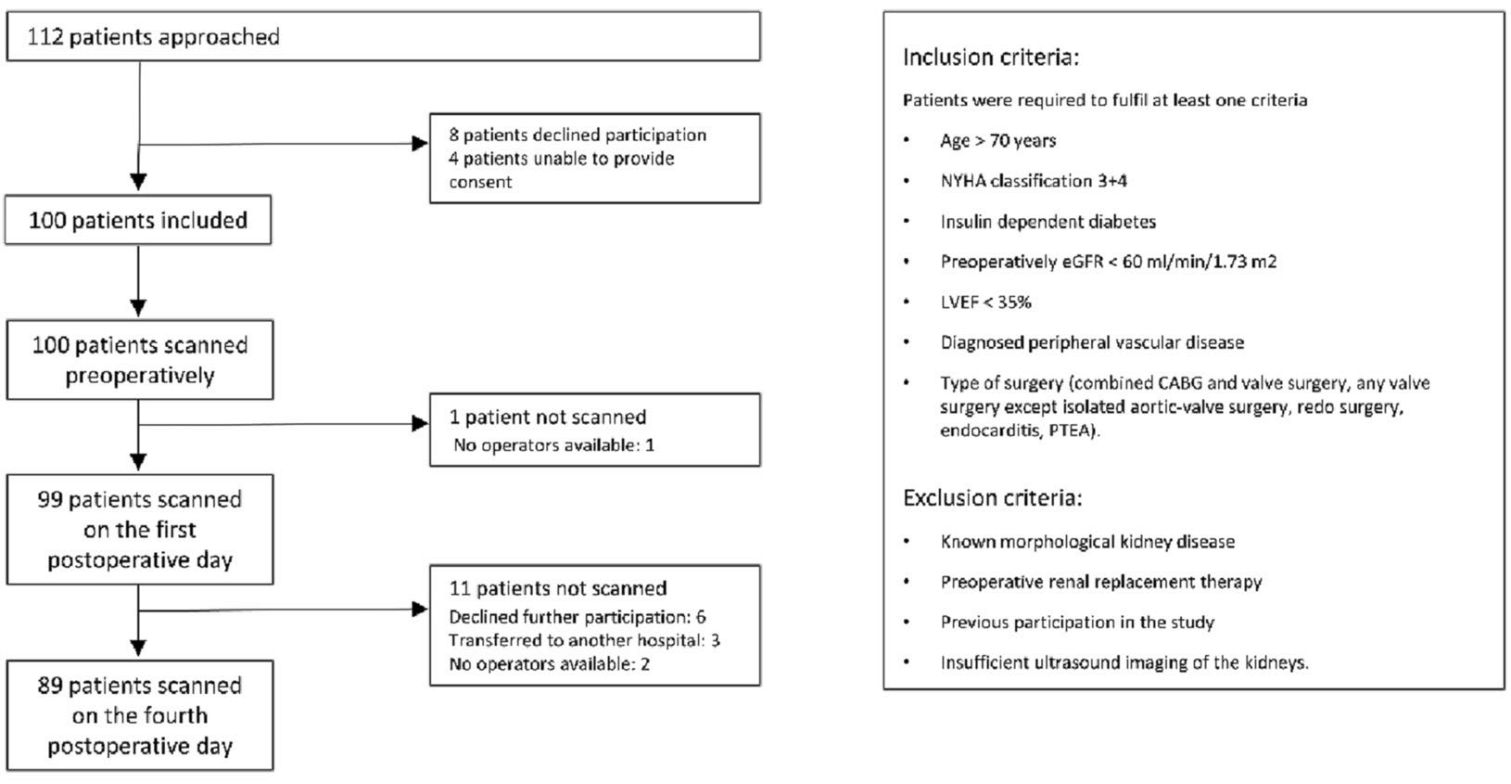
Flowchart of patients and ultrasound examinations in the study, including inclusion and exclusion criteria.

This was a prospective, observational, clinical study. Patients above 18 years of age undergoing elective open-heart, on-pump cardiac surgery at Aarhus University Hospital, Denmark with one or more known risk factors^3,12^ for postoperative AKI were eligible for inclusion. Risk factors are shown in Fig. 1 along with exclusion criteria.

The study was approved by the Central Denmark Region Committee on Health Research Ethics (identifier 1-10-72-267-18) registered at clinicaltrials.gov (identifier: NCT03727204, first posted 01.11.2018) as part of a two-centre multimodality study investigating the predictive capabilities and associations between different ultrasound measures and the development of AKI after cardiac surgery. All participants gave informed signed consent.

### Perioperative procedure

Patients were premedicated with paracetamol 1 g and diazepam 5 mg. Standard anaesthesia consisted of propofol, sufentanil and rocuronium. Cefuroxime and gentamycin were administered upon induction of anaesthesia although patients with preoperative renal impairment did not receive gentamycin as a standard routine. Procedures were conducted with cold blood cardioplegia and closed-circuit cardiopulmonary bypass with a targeted flow of 2.4 L/m^2^/min. As a rule, normothermia was maintained for CABG and aortic valve surgery during cardiopulmonary bypass; for mitral valve surgery, patients were cooled to 33° Celsius. Pulmonary thromboendarterectomy was performed in circulatory arrest at 18° Celsius. All decisions regarding patient management were at the discretion of the treating personnel and the investigators were not involved in the clinical care of the patients.

### Study timeline

On the last weekday before surgery, a baseline ultrasound examination was performed, blood samples were taken, and demographic patient characteristics were obtained. The ultrasound examination was repeated in the morning of the first postoperative day and on the fourth postoperative day. On the first postoperative day, fluid balance and invasive blood pressure measurements were obtained simultaneously with the ultrasound examination. Plasma creatinine was measured daily after surgery up to and including the fourth postoperative day for accurate determination of AKI development.

### Ultrasound examination

Ultrasound examinations were performed by two operators with more than 5 years’ experience in performing Doppler ultrasound examinations. Ultrasound examinations were performed using a Vivid S6 or S70N system (GE Healthcare, Horten, Norway) using a 4C or C1–6 curvilinear probe (GE Healthcare) with simultaneous electrocardiogram recordings. Patients were placed in the supine position and rested for 5 min before the examinations. Image analysis was performed in Echopac (GE Healthcare) by a single observer blinded to outcome variables.

#### Renal Doppler ultrasound

The kidney with the best imaging quality was selected and imaged in the longitudinal axis. Pulsed wave Doppler curves of the interlobar vessels were recorded with a sample volume of 4 mm and values were averaged from three measurements. In case of atrial fibrillation, averages consisted of three index beats^13^.

##### Renal venous flow pattern

We defined normal renal venous flow pattern as a continuous flow throughout the cardiac cycle, allowing a brief interruption in flow at end-diastole. Abnormal renal venous flow pattern was defined as either biphasic or monophasic flow^14,15^ (Supplementary Fig. S1). De novo abnormal renal venous flow pattern was defined as normal renal venous flow pattern preoperatively and abnormal renal venous flow pattern on the first postoperative day.

##### Renal venous stasis index

Renal venous stasis index (RVSI) was calculated as: (index cardiac cycle time—venous flow time)/index cardiac cycle time^13^. RVSI was severely skewed from normality, as there were many zero values, and RVSI was consequently categorically transformed. We defined two groups; low RVSI (0–0.30) and high RVSI (0.31–1.00). The RVSI cut-off of 0.31 was chosen based on the receiver operating characteristics (ROC). A detailed description of this calculation is provided in Supplementary Fig. S2.

##### Resistive index

The renal arterial resistive index (RI) was calculated as: (maximum arterial flow velocity—minimum arterial flow velocity)/maximum arterial flow velocity (Supplementary Fig. S2).

#### Portal vein Doppler ultrasound

Images of the portal vein were recorded with the transducer close to the right midaxillary line and pulsed wave Doppler curves were obtained from the intrahepatic part of the portal vein close to the liver hilum with a sample volume of 4 mm.

##### Portal pulsatility fraction

The portal venous pulsatility fraction was calculated as: (maximum portal venous flow velocity − minimum portal venous flow velocity)/maximum portal venous flow velocity (Supplementary Fig. S1).

### Invasive measurements

We defined the pulse pressure index as (systolic blood pressure − diastolic blood pressure)/systolic blood pressure, analogous to calculations of organ-specific indices as described above. Systemic perfusion pressure was calculated as mean arterial blood pressure − central venous pressure.

### Acute kidney injury

Postoperative AKI was defined by change in plasma creatinine according to the Kidney Disease Improving Global Outcomes (KDIGO) criteria^16^, with follow up was restricted to the first four postoperative days. Plasma creatinine taken on the last weekday before surgery served as the baseline reference. Based on the maximum plasma creatinine within the first four postoperative days, AKI was further categorised into AKI severity levels; mild AKI (KDIGO stage 1 AKI) and severe AKI (KDIGO stage 2 and 3 AKI). AKI stages 2 and 3 were combined into one group due to a low number of cases. We chose to distinguish between mild and severe AKI as the consequences of severe AKI are considerably more deleterious^4^. Further, major adverse kidney events (MAKE30) was recorded and defined as persistent renal dysfunction (creatinine ≥ 200% of baseline value), new-onset of haemodialysis or death, censored at first discharge or after 30 days of admission^17,18^.

### Clinical variables

Demographic and baseline clinical data were collected from the electronic patient record (MidtEPJ Columna, Systematic, Aarhus, Denmark) and The European System Operative Score Risk Evaluation score (EuroSCORE II) was calculated^19^.

Durations of anaesthesia, surgery, cardiopulmonary bypass, aortic cross-clamping and intensive care unit stay were retrieved from an electronic patient data management system (PDM, Critical Care Manager 8.6 (Picis Clinical Solutions, Wakefield, USA)). Invasive haemodynamic values were automatically recorded in the PDM system. Cumulative fluid balance, including urine output and bleeding, were manually recorded in the PDM system in real time by the anaesthesia or nursing staff until patient discharge from the ICU. Details on fluid balance were censored at 06.00 AM on the first postoperative morning. Use of mechanical ventilation and vasoactive medication was recorded at the time of the ultrasound examination on the first postoperative day. All data were stored in the REDCap electronic data capture tool^20^.

### Objectives

The primary objective was to examine the association between the renal venous flow pattern on the first postoperative day and the development of AKI from surgery until the fourth postoperative day. Secondary objectives were: (1) to find the optimal threshold values for venous and arterial Doppler flow indices for predicting the development of postoperative AKI, (2) to examine associations between perioperative RI, RVSI and portal pulsatility fraction and the development of AKI, (3) to assess the ability of a composite measure combining information of renal arterial and venous flow in predicting the development of AKI and (4) to describe associations between invasive blood pressures measured on the first postoperative day and the development of AKI.

### Statistical analysis

We expected a proportion of abnormal renal venous flow pattern on the first postoperative day of 30%^14^ and an AKI incidence of 30%^3^, given the inclusion criteria selecting patients at high risk. Assuming an odds ratio (OR) of 3.5 for AKI in patients with abnormal renal venous flow, we needed to include 99 patients (alpha = 0.05, power 0.8).

Patient and clinical characteristics were compared using one-way ANOVA or the Kruskal–Wallis test for continuous variables when appropriate and the χ^2^ test for dichotomous variables. We used a univariate logistic regression to test the associations between ultrasound measurements and the development of any degree of AKI and, secondarily, mild and severe AKI separately. We performed a multivariate logistic regression with adjustment for EuroSCORE II (expressing preoperative risk) and CPB time (expressing surgical burden) to test if ultrasound indices were independently associated with development of AKI. For the adjusted analysis we combined mild and severe AKI into one group due to the low number of cases with severe AKI.

The optimal thresholds for continuous renal ultrasound measurements on the first postoperative day was determined using ROC analyses. ROC analyses were performed for both correctly classifying no AKI versus mild/severe AKI, and, in addition, no AKI/mild AKI versus severe AKI. To include both arterial and venous flow variables in the prediction of AKI, a new variable reflecting both arterial and venous flow was created using RVSI and RI. The thresholds for RVSI and RI used in the combined analysis were the same as determined in the individual analyses.

20 patients were randomly (randomizer.com) selected for interobserver analysis of all ultrasound measurements at all three time points. Interobserver variability of continuous variables were analysed in accordance with the Bland Altman approach^21^. For categorical variables, reproducibility was assessed with the percentage of agreement and Cohen’s Kappa.

Patient characteristics are presented as medians (interquartile range (IQR)) for continuous values, and proportion (percentage) for categorical values. Ultrasound data are presented as means with 95% confidence interval (95% CI). Data were analysed using STATA 15 (StataCorp TX, USA) and a two-sided P-value < 0.05 was considered statistically significant.

### Ethics approval and consent to participate

The study was approved by the Central Denmark Region Committee on Health Research Ethics (identifier 1-10-72-267-18) and conducted in accordance with the Helsinki II declaration. All participants gave informed signed consent.

## Results

We enrolled 100 patients between October 2018 and December 2019 (Fig. 1). Baseline characteristics are displayed in Table 1. During the first four postoperative days, 33 patients developed AKI of which 25 developed mild AKI and eight developed severe AKI. No patients died within the first 30 postoperative days. Four patients, all from the severe AKI group, fulfilled the MAKE30 criteria. A preoperative ultrasound examination was performed in all patients; a single ultrasound examination on the first postoperative day and 11 ultrasound examinations on the fourth postoperative day could not be performed (Fig. 1). Data on admission, surgical details and clinical characteristics of the patients on the first postoperative day can be found in supplementary material (Supplementary Table S1).

**Table 1:** Baseline patient characteristics. Median and interquartile range (IQR) or number (n) and percent.

	No AKI (n = 67)	Mild AKI (n = 25)	Severe AKI (n = 8)	P-value
Age, years	72 (64; 75)	73 (70; 77)	62 (61; 70)	0.024*
Male, n	53 (79%)	21 (84%)	5 (63%)	0.66
Height (m)	1.73 (1.68; 1.79)	1.72 (1.65; 1.76)	1.72 (1.63; 1.78)	0.29
Weight (kg)	80.3 (74.0; 93.1)	80.7 (72.8; 89.1)	77.4 (70.7; 86.5)	0.57
BMI (kg/m^2^)	27.5 (24.9; 30.8)	28.5 (25.3; 30.3)	27.5 (23.7; 30.2)	0.93
**Surgery**				
CABG, n	33 (49%)	12 (48%)	1 (13%)	0.06
Aortic valve, n	6 (9%)	3 (12%)	1 (13%)	
Mitral valve, n	6 (9%)	4 (16%)	2 (25%)	
Two procedures, n	17 (25%)	4 (16%)	3 (38%)	
PTEA, n	5 (7%)	2 (8%)	0 (0%)	
Thoracic aortic surgery, n	0 (0%)	0 (0%)	1 (13%)	
Active endocarditis, n	0 (0%)	2 (8%)	0 (0%)	0.048*
Re-do surgery, n	5 (7%)	4 (16%)	5 (63%)	< 0.001*
**Co-morbidities**				
EuroSCORE II	1.58 (1.13; 2.00)	2.54 (1.48; 3.90)	3.60 (2.90; 4.82)	< 0.001*
Hypertension, n	47 (70%)	17 (68%)	7 (88%)	0.55
Diabetes mellitus				0.50
No, n	54 (81%)	18 (72%)	6 (75%)	
NIDDM, n	7 (10%)	4 (16%)	0 (0%)	
IDDM, n	6 (9%)	3 (12%)	2 (25%)	
Smoking				0.96
Present, n	10 (15%)	4 (16%)	1 (13%)	
Previous, n	17 (25%)	7 (28%)	3 (38%)	
Never, n	40 (60%)	14 (56%)	4 (50%)	
Hypercholesterolemia, n	39 (58%)	15 (60%)	3 (38%)	0.50
Ischemic heart disease, n	36 (54%)	13 (52%)	3 (38%)	0.69
Myocardial infarction < 90 days, n	15 (22%)	5 (20%)	0 (0%)	0.33
Known peripheral artery disease, n	4 (6%)	4 (16%)	0 (0%)	0.20
eGFR < 60 ml/min/1.73 m^2^, n	13 (19%)	14 (56%)	0 (0%)	< 0.001*
NYHA 3–4, n	4 (6%)	3 (12%)	2 (25%)	0.17
Left ventricular ejection fraction				0.43
> 50%, n	47 (70%)	16 (64%)	4 (50%)	
35–50%, n	15 (22%)	6 (24%)	4 (50%)	
< 35%, n	5 (7%)	3 (12%)	0 (0%)	
Pulmonary hypertension, n	9 (13%)	7 (28%)	1 (13%)	0.24
COPD, n	4 (6%)	6 (24%)	2 (25%)	0.031*
BMI > 35, n	7 (10%)	4 (16%)	0 (0%)	0.44

P-values refer to difference between any of two of groups no AKI, mild AKI and severe AKI. No AKI, mild AKI and severe AKI refer to patients who did not develop AKI, developed mild AKI (KDIGO grade 1) or developed severe AKI (KDIGO grades 2 + 3) within the first 4 postoperative days. * indicates statistically significant difference (P < 0.05).

*AKI* acute kidney injury, *BMI* body mass index, *CABG* coronary artery bypass grafting, *COPD* chronic obstructive pulmonary disease, *eGFR* estimated glomerular filtration rate, *IDDM* insulin dependent diabetes mellitus, *KDIGO* kidney disease improving global outcomes, *NIDDM* non-insulin dependent diabetes mellitus, *NYHA* New York Heart Association, *PTEA* pulmonary thromboendarterectomy.

### Ultrasound measurements

#### Renal venous flow pattern

**Figure 2:**
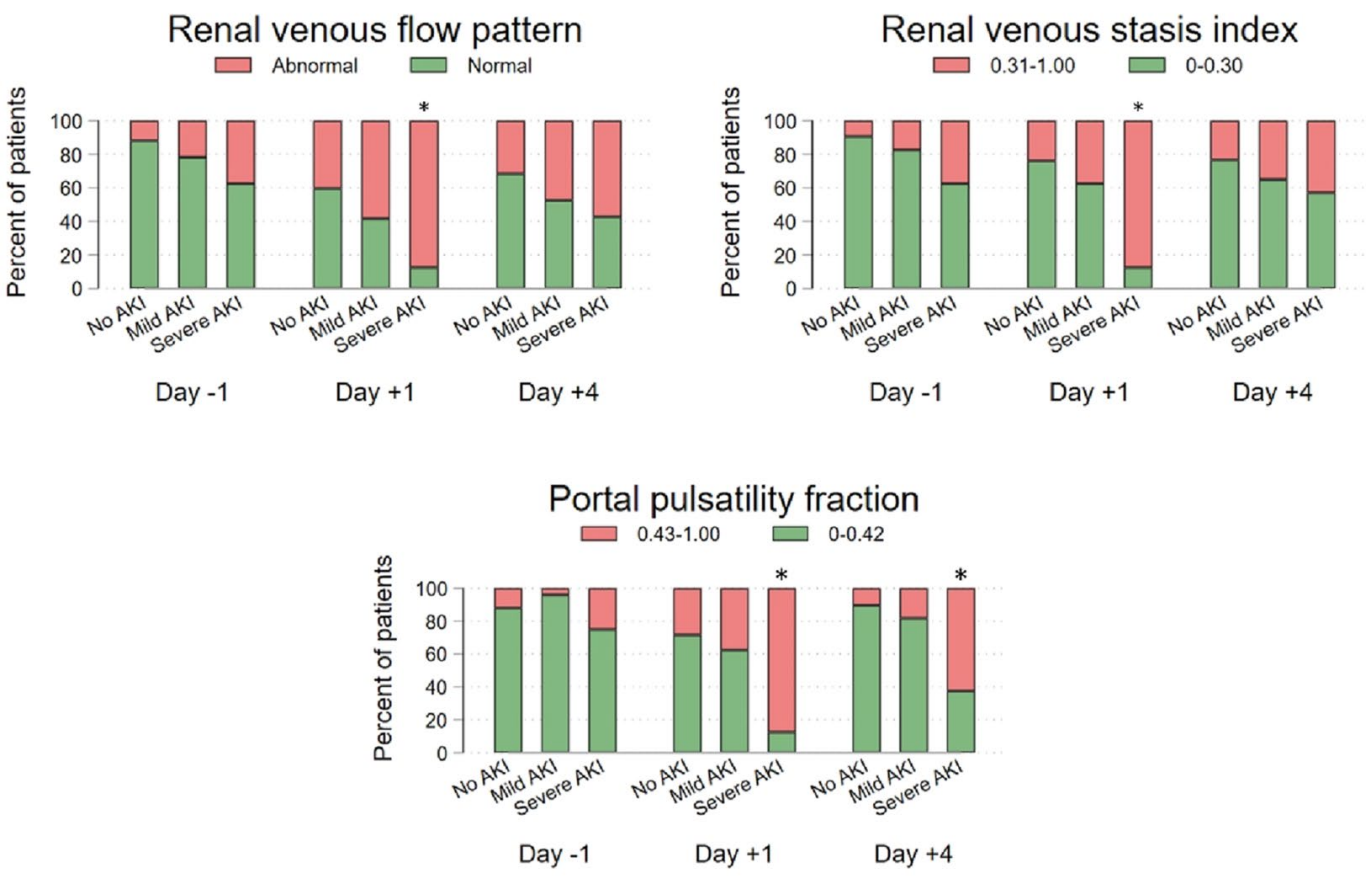
Distribution of patients within each of the venous ultrasound measurements in per cent according to different time points. Patients were divided according to the degree of AKI. Day − 1, + 1 and + 4 refer to the day before surgery, the first postoperative day and the fourth postoperative day. No AKI, mild AKI and severe AKI refer to patients who did not develop AKI, developed mild AKI (KDIGO grade 1) or developed severe AKI (KDIGO grades 2 + 3) within the first four postoperative days. *indicates statistically significant difference (P < 0.05) when comparing patients with no AKI and patients with either mild or severe AKI. See Table s1, supplementary material for exact values. *AKI* acute kidney injury, *KDIGO* kidney disease improving global outcomes.

On the first postoperative day, the renal venous flow pattern was abnormal in 14 of 24 (59%) patients who developed mild AKI, and in seven out of the eight (88%) patients with severe AKI (Fig. 2). Renal venous flow pattern was abnormal in 16 patients preoperatively. In the remaining 84 patients, 37 (44%) patients had de novo abnormal renal venous flow pattern on the first day, including 10 out of 19 (53%) who developed mild AKI and five out of five (100%) who developed severe AKI (Fig. 2).

Patients with an abnormal renal venous flow pattern on the first postoperative day were more likely to develop any degree of AKI (odds ratio (OR) 2.83 (95% CI 1.18; 6.80), P = 0.020) (Table 2) and the association was also present when looking at severe AKI only (OR 8.54 (95% CI 1.01; 72.2), P = 0.046) (Table 3). In the adjusted analysis abnormal renal venous flow pattern was not associated with any degree of AKI (adjusted OR (aOR) 1.69 (95% CI 0.60; 4.80), P = 0.32). Renal venous flow patterns were not significantly associated with AKI preoperatively or on the fourth postoperative day (all P-values ≥ 0.06). The distribution of renal venous flow pattern at the different time points among patients with no, mild or severe AKI are shown in Fig. 2. The negative predictive value for a normal renal venous flow pattern was 98.0% (95% CI 89.6%; 100%) for severe AKI, 78.4% (95% CI 64.7%; 88.7%) for any degree of AKI and 100% (95 CI 93.0%; 100%) for MAKE30 (Table 4, Supplementary Table S2).

**Table 2:** Univariate and multivariate logistic regression models for the odds ratio (OR) of developing AKI after cardiac surgery according to studied variables on the first postoperative day.

Ultrasound indices 1st postoperative day	Univariate analyses			Multivariate analyses		
	OR	95% CI	P-value	OR	95% CI	P-value
**Renal venous flow pattern**						
Normal	1.0 (Ref)			1.0 (Ref)		
Abnormal	2.83	(1.18; 6.80)	0.020*	1.69	(0.60; 4.80)	0.32
**RVSI**						
Low (0–0.30)	1.0 (Ref)			1.0 (Ref)		
High (0.31–1.00)	3.19	(1.31; 7.78)	0.011*	1.70	(0.58; 4.94)	0.33
Resistive index	1.21	(1.10; 1.34)	< 0.001*	1.23	(1.09; 1.40)	0.001*
Portal pulsatility fraction	1.02	(1.00; 1.05)	0.08	1.01	(0.98; 1.03)	0.55

Multivariate logistic regression adjusted for cardiopulmonary bypass (CPB) time and EuroSCORE II. OR, 95% CI (confidence interval) and P-value for developing any degree of AKI after cardiac surgery with no AKI as reference group. All values except EuroSCORE II refer to the first postoperative day. Renal venous flow pattern and renal venous stasis index (RVSI) are treated as categorical variables. Other variables are treated as continuous variables. Higher OR depicts higher risk of AKI.  * Indicates statistically significant difference (P < 0.05).

*AKI* acute kidney injury, *CPB* cardiopulmonary bypass.

**Table 3:** Univariate logistic regression.

	Time	Mild AKI (n = 25)			Severe AKI (n = 8)		
		OR	95% CI	P-value	OR	95% CI	P-value
**Abnormal renal venous flow pattern**							
Compared to normal renal venous flow pattern	− 1	2.06	(0.58; 7.32)	0.26	3.5	(0.73; 16.6)	0.06
	+ 1	2.07	(0.80; 5.35)	0.13	8.54	(1.01; 72.2)	0.046*
	+ 4	1.96	(0.67; 5.70)	0.22	2.41	(0.50; 11.6)	0.27
**RVSI (0.31–1.00)**							
Compared to RVSI (0–0.30)	− 1	2.06	(0.50; 8.51)	0.32	4.53	(0.92; 22.3)	0.06
	+ 1	1.91	(0.70; 5;19)	0.20	18.48	(2.16; 158)	0.008*
	+ 4	1.78	(0.59; 5.40)	0.31	2.10	(0.43; 10.2)	0.36
**Portal pulsatility fraction**							
Per 0.01 increase	− 1	0.99	(0.95; 1.03)	0.60	1.02	(0.96; 1.09)	0.50
	+ 1	1.00	(0.97; 1.03)	0.88	1.07	(1.02; 1.13)	0.005*
	+ 4	1.02	(0.98; 1.06)	0.38	1.06	(1.01; 1.12)	0.021*
**Resistive index**							
Per 0.01 increase	− 1	1.02	(0.96; 1.08)	0.49	1.02	(0.71; 1.11)	0.71
	+ 1	1.19	(1.08; 1.32)	0.001*	1.17	(1.02; 1.35)	0.023*
	+ 4	1.04	(0.95; 1.12)	0.41	1.02	(0.90; 1.15)	0.74
**Mean arterial pressure**							
Lower MAP increases OR	+ 1	1.05	(0.90; 1.01)	0.09	1.15	(1.02; 1.29)	0.022*
**Central venous pressure**							
Higher CVP increases OR	+ 1	1.00	(0.87; 1.16)	0.93	1.91	(1.23; 2.96)	0.004*
**Systemic perfusion pressure**							
Lower SPP increases OR	+ 1	1.04	(0.99; 1.10)	0.10	1.24	(1.07; 1.43)	0.003*
**Pulse pressure**							
Lower PP increases OR	+ 1	1.04	(1.01; 1.08)	0.017 *	1.05	(0.99; 1.10)	0.10
**Pulse pressure index**							
Lower PPI increases OR	+ 1	1.05	(0.99; 1.10)	0.10	1.02	(0.94; 1.11)	0.58

Odds ratio (OR), 95% CI (confidence interval) and P-value for developing mild AKI and severe AKI, respectively after cardiac surgery with no AKI as reference group.

The numbers − 1, + 1 and + 4 refer to the time of the ultrasound examination at the day before surgery, the first postoperative day and the fourth postoperative day. Abnormal venous flow pattern and renal venous stasis index (RVSI) are treated as dichotomous variables and resistive index portal pulsatility fraction and hemodynamic values are treated as continuous variables. Higher OR depicts higher risk of mild or severe AKI. No AKI, mild AKI and severe AKI refers to patients who did not develop AKI, developed mild AKI (KDIGO grade 1) or developed severe AKI (KDIGO2 + 3) within the first four postoperative days.  * Indicates statistically significant difference (P < 0.05).

**Table 4:** Receiver operating characteristics (ROC) of the studied ultrasound indices on the first postoperative day in relation to correctly classifying patients with any degree of AKI (KDIGO stage 1 + 2 + 3) (upper panel) and severe AKI (KDIGO stage 2 + 3) in the lower panel.

Ultrasound parameter and threshold—1st postoperative day	Sensitivity (95% CI)	Specificity (95% CI)	PPV (95% CI)	NPV (95% CI)	LR+	LR−	AUC (95% CI)
**Any degree of AKI (n = 33)**							
Abnormal renal venous flow pattern	65.6 (46.8; 81.4)	59.7 (47.0; 71.5)	43.8 (29.5; 58.8)	78.4 (64.7; 88.7)	1.63	0.576	0.63 (0.52; 0.73)
De novo abnormal renal venous flow pattern	58.3 (36.6; 77.9)	61.7 (48.2; 73.9)	37.8 (22.5; 55.2)	78.7 (64.3; 89.3)	1.52	0.676	0.60 (0.48; 0.72)
RVSI ≥ 0.31	50.0 (31.9; 68.1)	76.1 (64.1; 85.7)	50.0 (31.9; 68.1)	76.1 (64.1; 85.7)	2.09	0.657	0.63 (0.53; 0.73)
RI ≥ 0.73	87.5 (71.0; 96.5)	58.2 (45.5; 70.2)	50.0 (36.3; 63.7)	90.7 (77.9; 97.4)	2.09	0.215	0.73 (0.65; 0.81)
Portal pulsatility fraction ≥ 0.43	50.0 (31.9; 68.1)	71.6 (59.3; 82.0)	45.7 (28.8; 63.4)	75.0 (62.6; 85.0)	1.76	0.698	0.61 (0.51; 0.71)
RI < 0.73 and RVSI < 0.31	90.6 (75.0; 98.0)	46.3 (34.0; 58.9)	44.6 (32.2; 57.5)	91.2 (76.3; 98.1)	1.69	0.203	0.68 (0.60; 0.76)
RI ≥ 0.73 and RVSI ≥ 0.31	46.9 (29.1; 65.3)	88.1 (77.8; 94.7)	65.2 (42.7; 83.6)	77.6 (66.6; 86.4)	3.93	0.603	0.68 (0.56; 0.77)
**Severe AKI (n = 8)**							
Abnormal renal venous flow pattern	87.5 (47.3; 99.7)	54.9 (44.2; 65.4)	14.6 (6.1; 27.8)	98.0 (89.6; 100)	1.94	0.228	0.71 (0.58; 0.85)
De novo abnormal renal venous flow pattern	100 (47.8; 100)	59.5 (47.9; 70.4)	13.5 (4.5; 28.8)	100 (92.5; 100)	2.47	0	0.80 (0.74; 0.85)
RVSI ≥ 0.31	87.5 (47.3; 99.7)	72.5 (62.2; 81.4)	21.9 (9.3; 40.0)	98.5 (92.0; 100)	3.19	0.172	0.80 (0.67; 0.93)
RI ≥ 0.73	87.5 (47.3; 99.7)	46.2 (35.6; 56.9)	12.5 (5.2; 24.1)	97.7 (87.7; 99.9)	1.63	0.271	0.67 (0.54; 0.80)
Portal pulsatility fraction ≥ 0.43	87.5 (47.3; 99.7)	69.2 (58.7; 91.5)	20.0 (8.4; 36.9)	98.4 (91.6; 100)	2.84	0.181	0.78 (0.65; 0.92)
RI < 0.73 and RVSI < 0.31	100 (63.1; 100)	37.4 (27.4; 48.1)	12.3 (5.5; 22.8)	100 (89.7; 100)	1.57	0	0.69 (0.64; 0.74)
RI ≥ 0.73 and RVSI ≥ 0.31	75.0 (34.9; 96.8)	81.3 (71.8; 88.7)	26.1 (10.2; 48.4)	97.4 (90.8; 99.7)	4.01	0.307	0.78 (0.62; 0.95)

De novo abnormal renal venous flow pattern was defined as normal pattern preoperatively and abnormal renal venous flow pattern on the first postoperative day.

*AKI* acute kidney injury, *AUC* area under the curve, *CI* confidence interval, *KDIGO* Kidney Disease Improving Global Outcomes, *LR* + positive likelihood ratio, *LR* − negative likelihood ratio, *NPV* negative predictive value, *PPV* positive predictive value, *RI* resistive index, *RVSI* renal venous stasis index.

#### Renal venous stasis index

High RVSI values on the first postoperative day were significantly associated with developing any degree of AKI, (OR 3.19 (95% CI 1.31; 7.78), P = 0.011) (Table 2) with a corresponding OR for severe AKI of 18.48 (95% CI 2.16; 158, P = 0.008) (Table 2). RVSI was not significantly associated with AKI in the adjusted analysis (Table 2). There were no significant differences between AKI groups at other time points, regardless of AKI severity (all P ≥ 0.06) (Table 3). When RVSI was above the threshold of 0.31 on the first postoperative day, the sensitivity for detecting patients severe AKI was 87.5% (95% CI 47.3%; 99.7%) (Table 4).

#### Resistive index

RI on the first postoperative day was significantly associated with both mild (OR 1.19 (95% CI 1.08; 1.32), P = 0.001) and severe AKI (OR 1.17 (95% CI 1.02; 1.35), P = 0.023) (Table 3). RI was also significantly associated with AKI in the adjusted analysis (aOR 1.23 (95% CI 1.09; 1.40), P = 0.001) (Table 2). There were no significant associations at other time points (P ≥ 0.41). Using a threshold of 0.73, the sensitivity of RI for any degree of AKI was 87.5% (95% CI 71.0%; 96.5%). (Table 4).

#### Portal pulsatility fraction

Portal pulsatility fraction on the first postoperative day was not associated with any degree of AKI (OR 1.02 (95% CI 1.00; 1.05), P = 0.08) (Table 2). However, when AKI was subdivided, portal pulsatility fraction was significantly associated with severe AKI (OR: 1.07 (95% CI 1.02; 1.13), (P = 0.005)), but not with mild AKI (P = 0.88) (Table 3). In the adjusted analysis, portal pulsatility fraction on the first postoperative day was not associated with AKI (Table 2). The sensitivity of portal pulsatility fraction ≥ 0.43 was 87.5% (95% CI 47.3%; 99.7%) for severe AKI and 50.0% (95% CI 31.9%; 68.1%) for any degree of AKI (Table 4). The corresponding negative predictive value was 98.4% (95% CI 91.6%; 100%) for severe AKI and 75.0% (95% CI 62.6%; 85.0%) for any degree of AKI.

#### RVSI and RI combined

The negative predictive of value of RVSI and RI threshold values combined for severe AKI was 100% (95% CI 89.7; 100) (RI < 0.73 and RVSI < 0.31) with a corresponding positive likelihood ratio of 4.01. The positive predictive value for severe AKI was 26.1% (95% CI 10.2%; 48.8%) (Table 4). The predictive values for MAKE30 are provided in Supplementary Table S2.

#### Invasive blood pressures

On the first postoperative day, lower mean arterial pressure was associated with severe AKI (OR 1.15 (95% CI 1.02; 1.29), P = 0.022), but not mild AKI (P = 0.09) (Table 3). Higher central venous pressure was significantly associated with severe AKI (OR 1.91 (95% CI 1.23; 2.96), P-value = 0.004), but not with mild AKI (P = 0.93). Furthermore, lower systemic perfusion pressure was associated with severe AKI (OR 1.24 (95% CI 1.07; 1.43), P-value = 0.003), but not with mild AKI (P-value = 0.10). Pulse pressure index was not associated with mild or severe AKI (P-values ≥ 0.10).

#### Interobserver variation of ultrasound measurements

The interobserver agreement for renal venous flow pattern was 92.5% with a Kappa value of 0.84. For RVSI, interobserver agreement was 87.8% (Kappa value 0.67). The mean interobserver variation was − 0.2% (95% CI − 2.0% to 1.6%, 95% limits of agreement: − 9.2% to 8.8%) for RI and − 1.3% (95% CI − 8.8% to 6.3%, 95% limits of agreement: − 18.5% to 16.1%) for portal pulsatility fraction.

## Discussion

We found that renal venous flow pattern, RVSI, RI and portal pulsatility fraction measured on the first postoperative day were associated with postoperative AKI of any degree after cardiac surgery. Furthermore, when AKI was divided in mild and severe cases, renal venous flow pattern, RVSI and portal pulsatility were only significantly associated with severe AKI.

Our results suggest that the effects on renal Doppler flow measurements were primarily caused by changes in right-sided venous flow rather than organ-specific changes to the kidneys. First, we found similar diagnostic abilities of renal- and portal indices for predicting AKI. This suggests that the degrees of venous congestion within and outside the renal capsule were comparable. If localised interstitial renal oedema rather than systemic venous congestion was dominant in determination of the Doppler venous flow, we would have expected venous flow alterations to be more pronounced in the renal veins than in the portal vein. Further, the associations between AKI and venous ultrasound indices had the same pattern as the associations between AKI and other variables related to systemic venous congestion; central venous pressure, fluid balance and weight balance (Supplementary Table S1). This is in accordance with a study that demonstrated that fluid infusion and subsequent diuretic treatment in patients with heart failure induced changes in renal ultrasound measurements compatible with induction and relief of renal congestion^22^.

Renal venous stasis index has been introduced as a novel modality, to measure the temporal continuum of renal congestion with a continuous index. As RVSI was heavily skewed with many zero values and we thus categorically transformed RVSI. Following this, associations between both renal venous flow category as well as RVSI categories and the development of AKI were comparable. RVSI did not show superior performance compared to renal flow category, we suggest that renal venous flow pattern can be used primarily as this is simple to assess without the need for quantification, which favours its clinical applicability.

RI was the most sensitive ultrasound measurement for identifying patients with any degree of AKI. Furthermore, the high negative predictive value of RI favours its use as a marker of normal postoperative renal function. The challenging aspect of using RI in a clinical context is that values are often found within a fairly narrow range. Minor uncertainties in measurements may have a relatively large impact on whether a result is above or below a given threshold, stressing the need for meticulous Doppler measurements.

In a novel approach, we combined both arterial and venous Doppler measurements for predicting AKI. Postoperative values of both RI and RVSI above threshold values produced high positive likehood ratios and the specificities for mild and severe AKI and MAKE30 were high. From a clinical perspective, patients who develop AKI are thus likely to have RVSI and RI above thresholds of 0.31 and 0.73, respectively. As the positive predictive value for AKI was moderate, the measurements can be used as early markers of concurrent or impending AKI, allowing for timely interventions. Conversely, the combination of low RI and low RVSI makes AKI very unlikely. In general, the negative predictive values for all ultrasound Doppler indices for severe AKI and MAKE30 were very high. Therefore, regardless of ultrasound index, a normal value on the first postoperative day effectively rules out development of severe AKI after cardiac surgery. MAKE30 has been recommended as an endpoint in clinical trials with AKI. It identifies patients with persistent renal failure, which is an important prognostic factor after cardiac surgery^23,24^. It is thus important to identify these patients in the postoperative period, for which Doppler indices of renal perfusion can be of use.

Preoperative renal or portal vein Doppler measurements were not significantly associated with the development of AKI. Among the patients who developed severe AKI and had normal preoperative renal venous flow pattern, all had de novo abnormal flow on the first postoperative day. In general, preoperative renal and portal vein ultrasound measurements are probably of limited clinical value. In contrast, measurements performed on the first postoperative day provided important information supplementing other clinical data for clinical and individual estimation of the risk for developing severe AKI.

The renal and portal venous ultrasound indices were not independently associated with AKI after adjusting for EuroSCORE II and CPB time. This contradicts previous findings^14^ and can be partly explained as the renal and portal venous indices in the present study were particularly associated with severe but not mild AKI. In the adjusted regression analysis, all AKI cases were pooled as the number of cases with severe AKI was low, and this may have diluted the association between AKI and venous Doppler measurements.

We found that reduced systemic perfusion pressure was associated with development of AKI, confirming previous studies^25,26^. Further, a recent study of patients in the ICU found that abnormalities in renal and portal venous Doppler measurements were associated with major adverse kidney events^15^. Hence, a clinical approach to selected patients at high risk of severe AKI should include evaluation of venous conditions, and this can be done with point-of-care ultrasound. In substantiation of this argument, severe disturbances in renal and portal venous flow were recently shown to outperform central venous pressure as an early marker of AKI in patients after cardiac surgery^27^.

The present study has limitations. The number of AKI cases restricted the number of variables to be included in the adjusted regression analysis, which may have introduced residual confounding. There was an uneven distribution of patients that received norepinephrine or who were mechanically ventilated on the first postoperative day (Table 3), both being more frequent in the severe AKI group. However, we believe these factors constitute a part of the AKI causal pathway. The effect of individual ventilator settings and changes in afterload on renal and portal Doppler measurements in patients undergoing cardiac surgery remain to be described, but it is well knowing that determinants of cardiac function depend on ventilator settings^28^. Further, this study does not address the effects of interventions on renal Doppler flow measurements. We suggest that future trials test a strategy for normalising renal and portal venous flow measurements in prevention or treatment of AKI in patients with concurrent signs of venous congestion.

## Conclusion

Ultrasound Doppler indices of renal and portal venous congestion on the first postoperative day were significantly associated with the development of AKI after cardiac surgery, in particular severe AKI. RI was independently associated with both mild and severe AKI. Renal and portal Doppler ultrasonography can be used to identify patients at high risk of AKI after cardiac surgery. Further, if both RI and venous flow were normal, the risk of developing AKI was very low. Point-of-care Doppler ultrasound measurements can provide clinicians with insight into the individual patient’s degree of venous congestion and support prediction of AKI after cardiac surgery.

## Supplementary Information

Supplementary Information.

## Data Availability

The datasets used and analysed during the current study are available from the corresponding author on reasonable request.
