# Supplementary material for: Perioperative Doppler measurements of renal perfusion are associated with acute kidney injury in patients undergoing cardiac surgery
Source: Sci Rep. 2021 Oct 5;11:19738. doi: 10.1038/s41598-021-99141-y (PMC8492663; doi:10.1038/s41598-021-99141-y)
Supplement: Supplementary file 1 — Supplementary Information. [file 41598_2021_99141_MOESM1_ESM.docx]

Supplementary Table 1:

|  | **No AKI (n=67)** |  | **Mild AKI (n=25)** |  | **Severe AKI (n=8)** |  | **P-value** |
| --- | --- | --- | --- | --- | --- | --- | --- |
| MAKE30, (n) | 0 (0) |  | 0 (0) |  | 4 (50) |  | < 0.001 * |
| Surgery time (min) | 207 (177 ; 244) |  | 224 (182 ; 255) |  | 356 (314 ; 418) |  | 0.001 * |
| CPB time (min) | 104 (83 ; 132) |  | 117 (88 ; 182) |  | 217 (129 ; 277) |  | 0.018 * |
| Cross-clamp time (min) | 65 (49 ; 89) |  | 76 (55 ; 102) |  | 99 (68 ; 142) |  | 0.07 |
| Bleeding (ml) | 770 (450 ; 1,040) |  | 950 (600 ; 1,200) |  | 1,905 (1,260 ; 4,065) |  | < 0.001 * |
| ICU time (hours) | 22 (19.5 ; 23.9) |  | 24.5 (22.5 ; 57.5) |  | 147.5 (81.5 ; 224) |  | < 0.001 * |
| Hospital stay (days) | 8 (6 ; 11) |  | 13.5 (9 ; 20) |  | 33 (25 ; 50) |  | < 0.001 * |
| Fluid balance day 1 (ml) | 1.037 (0.456 ; 1.656) |  | 1.089 (0.674 ; 1.410) |  | 2.781 (2.107 ; 3.470) |  | 0.002 * |
| Weight balance day 1 (kg) | 1.9 (1.0 ; 3.3) |  | 1.3 (0.7 ; 2.8) |  | 4.2 (1.9 ; 5.2) |  | 0.031 * |
| Diuresis – day 1, averaged after surgery, (ml/hour) | 83 (63 ; 109) |  | 85 (65 ; 107) |  | 58 (44 ; 79) |  | 0.044 * |
| Gentamycin given preoperatively, n | 56 (86) |  | 21 (84) |  | 8 (100) |  | 0.47 |
| NSAID given postoperatively, n | 47 (70) |  | 14 (56) |  | 1 (13) |  | 0.005 * |
| Mechanical ventilation |  |  |  |  |  |  |  |
| Yes – day 1, n | 6 (9) |  | 4 (16) |  | 7 (88) |  | 0.001 * |
| Yes – day 4, n | 0 (0) |  | 0 (0) |  | 4 (50) |  | < 0.001 * |
| **Medication 1^st^ postoperative day** |  |  |  |  |  |  |  |
| Norepinephrine, n | 10 (15%) |  | 10 (40%) |  | 8 (100%) |  | < 0.001 * |
| Furosemide, n | 1 (1%) |  | 4 (16%) |  | 5 (63%) |  | < 0.001* |
| Dobutamine, n | 5 (7%) |  | 3 (12%) |  | 1 (13%) |  | 0.75 |
| Milrinone, n | 0 (0%) |  | 2 (8%) |  | 2 (25%) |  | 0.001 * |
| **Haemodynamic variables  1^st^ postoperative day** |  |  |  |  |  |  |  |
| Mean arterial pressure (mmHg) | 81 (76 ; 87) |  | 78 (72 ; 84) |  | 72 (72 ; 74) |  |  |
| Pulse pressure (mmHg) | 65 (56 ; 77) |  | 58 (46 ; 65) |  | 54 (48 ; 66) |  |  |
| Pulse pressure index | 0.54 (0.47 ; 0.60) |  | 0.50 (0.44 ; 0.54) |  | 0.50 (0.44 ; 0.57) |  |  |
| Central venous pressure (mmHg) | 10 (8 ; 12) |  | 10 (8 ; 12) |  | 16 (13 ; 16) |  |  |
| Systemic perfusion pressure)  (mmHg) | 72 (65 ; 78) |  | 69 (64 ; 74) |  | 58 (57 ; 61) |  |  |
| Heart rate (beats∙min^-1^) | 78 (69 ; 81) |  | 80 (72 ; 89) |  | 81 (80 ; 90) |  |  |

Clinical patient characteristics. Median and interquartile range (IQR) or number (n) and per cent. P-values refer to difference between any of two of groups no AKI, mild AKI and severe AKI. Medications and haemodynamic variables are recorded at the time of the ultrasound examination on the first postoperative day. No AKI, mild AKI and severe AKI refer to patients who did not develop AKI, developed mild AKI (KDIGO stage 1) or developed severe AKI (KDIGO stage 2+3) within the first four postoperative days.

Abbreviations: AKI (acute kidney injury), CPB (cardiopulmonary bypass), ICU (intensive care unit), KDIGO (Kidney Disease Improving Global Outcomes), MAKE30 (Major adverse kidney events with 30 days),NSAID (non-steroidal anti-inflammatory drug).

Supplementary Table 2:

| **Ultrasound parameter  and threshold -**  **1^st^ postoperative day** | **Sensitivity (95% CI)** | **Specificity (95% CI)** | **PPV (95% CI)** | **NPV (95% CI)** | **LR +** | **LR −** | **AUC (95% CI)** |
| --- | --- | --- | --- | --- | --- | --- | --- |
|  | **MAKE30 (n=4)** | | | | | | |
|  |  |  |  |  |  |  |  |
| **Abnormal renal venous  flow pattern** | 100 (39.8 ; 100) | 53.7 (43.2 ; 64.0) | 8.3 (2.3 ; 20.0) | 100 (93.0 ; 100) | 2.16 | 0 | 0.77 (0.72 ; 0.82) |
|  |  |  |  |  |  |  |  |
| **De novo abnormal renal venous flow pattern** | 100 (15.8 ; 100) | 57.3 (45.9 ; 68.2) | 5.4 (0.6 ; 18.2) | 100 (92.5 ; 100) | 2.34 | 0 | 0.79 (0.73 ; 0.84) |
|  |  |  |  |  |  |  |  |
| **RVSI ≥ 0.31** | 100 (39.8 ; 100) | 70.5 (60.3 ; 79.4) | 12.5 (3.5 ; 29) | 100 (94.6 ; 100) | 3.39 | 0 | 0.85 (0.81 ; 0.90) |
|  |  |  |  |  |  |  |  |
| **RI ≥ 0.73** | 100 (39.8 ; 100) | 45.3 (35.0 ; 55.8) | 7.1 (2.0 ; 17.3) | 100 (91.8 ; 100) | 1.83 | 0 | 0.73 (0.68 ; 0.78) |
|  |  |  |  |  |  |  |  |
| **Portal pulsatility fraction ≥ 0.43** | 75 (19.4 ; 99.4) | 66.3 (55.9 ; 75.7) | 8.6 (1.8 ; 23.1) | 98.4 (91.6 ; 100) | 2.23 | 0.38 | 0.71 (0.46 ; 0.96) |
|  |  |  |  |  |  |  |  |
| **RI < 0.73 *and* RVSI < 0.31** | 100 (39.8 ; 100) | 35.4 (25.9 ; 45.8) | 6.1 (1.7 ; 14.8) | 100 (89.7 ; 100) | 1.55 | 0 | 0.68 (0.63 ; 0.73) |
|  |  |  |  |  |  |  |  |
| **RI ≥ 0.73 *and* RVSI ≥ 0.31** | 100 (39.8 ; 100) | 80.2 (70.8 ; 87.6) | 17.4 (5.0 ; 38.8) | 100 (95.3 ; 100) | 5.05 | 0 | 0.90 (0.86 ; 0.94) |
|  |  |  |  |  |  |  |  |

Receiver operating characteristics (ROC) of the studied ultrasound indices on the first postoperative day in relation to correctly classifying patients with major adverse kidney events within 30 days (MAKE30), defined as persistent renal dysfunction (creatinine ≥ 200% of baseline value), new-onset of haemodialysis or death, censored at first discharge or after 30 days of admission. De novo abnormal renal venous flow pattern was defined as normal pattern preoperatively and abnormal renal venous flow pattern on the first postoperative day.

Abbreviations: AUC (area under the curve), CI (confidence interval), LR+ (positive likelihood ratio), LR− (negative likelihood ratio), NPV (negative predictive value), PPV (positive predictive value), RI (resistive index), RVSI (renal venous stasis index).

Supplementary Figure 1:


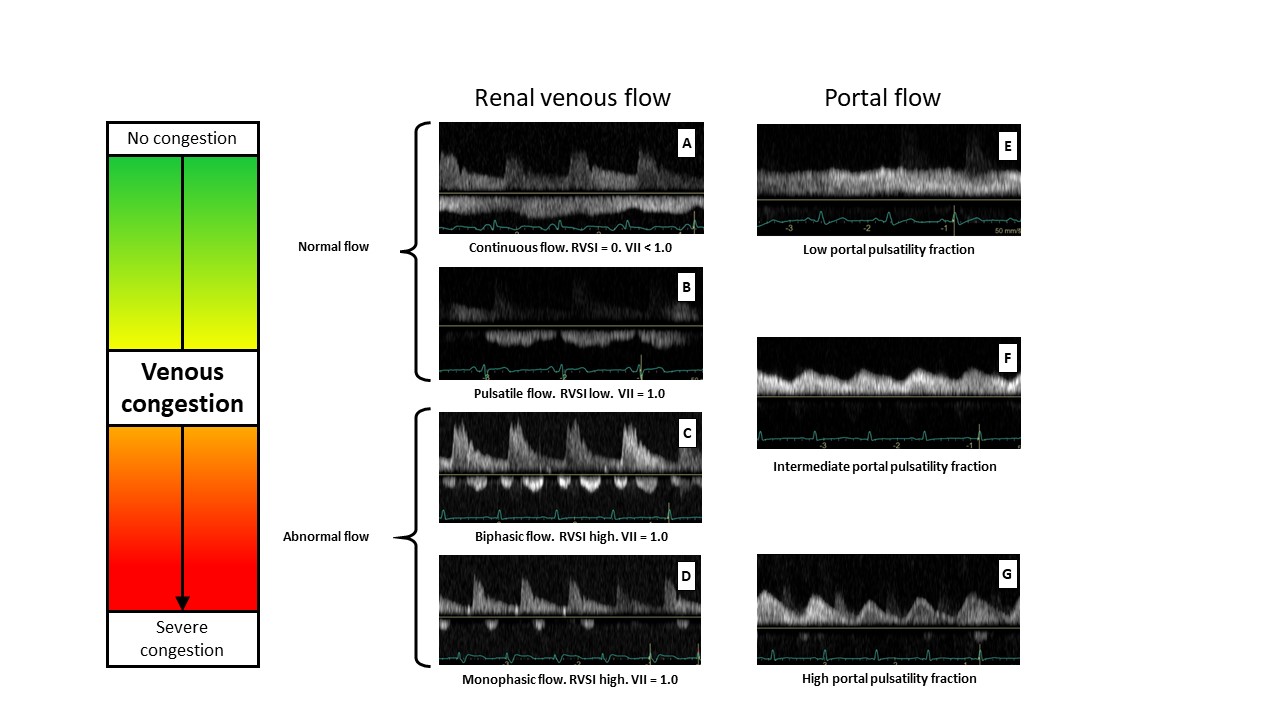


Doppler ultrasound examinations of the renal interlobar venous vessels (Figures A – D) and the portal vein (Figures E – G) in the proposed relationships with increasing venous congestion. In renal images (A–D), the venous flow is shown below the horizontal line and arterial flow is shown above the horizontal line. All ultrasound images are shown with simultaneously obtained electrocardiograms. A: Continuous renal venous flow through the entire cardiac cycle. B: Pulsatile renal venous flow with a short end-diastolic pause in venous flow, just after the p-wave in the electrocardiogram. C: Biphasic renal venous flow with two pauses in one cardiac cycle. D: Monophasic flow where renal venous flow only occurs in diastole. Normal venous flow included continuous flow and pulsatile flow patterns, abnormal flow included biphasic and monophasic flow patterns. E: Portal venous flow with low variation between maximum and minimum velocity, therefore a low portal pulsatility fraction. F: Increased difference between maximum and minimum velocity and intermediate portal pulsatility fraction. G: Large variation between maximum and minimum portal venous flow velocity, giving a high portal pulsatility fraction.

Supplementary Figure 2:


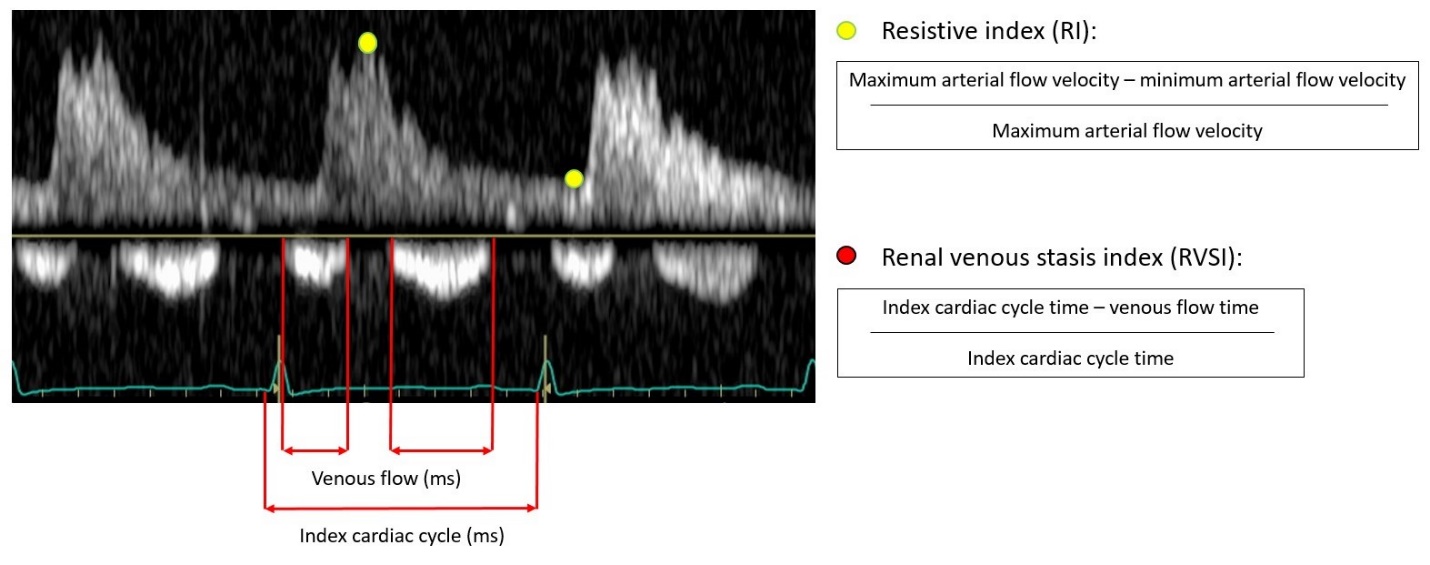


Doppler ultrasound tracing of the renal arterial and venous interlobar vessels and calculation of flow measures. The Doppler flow curve above the line is from the arterial vessel and the Doppler flow curve below the line is from the venous vessels. In the present patient, the venous flow was biphasic/abnormal, RI was 0.75 and RVSI was 0.40.
